# Oxygen as a Driver of Early Arthropod Micro-Benthos Evolution

**DOI:** 10.1371/journal.pone.0028183

**Published:** 2011-12-02

**Authors:** Mark Williams, Jean Vannier, Laure Corbari, Jean-Charles Massabuau

**Affiliations:** 1 Department of Geology, University of Leicester, Leicester, United Kingdom; 2 UMR 5276 CNRS, Laboratoire de Géologie de Lyon: Terre, Planètes, Environnement, Université Lyon 1, Villeurbanne, France; 3 UMR 7138 CNRS, Systématique, Adaptation, Evolution, Muséum National d'Histoire Naturelle, Paris, France; 4 UMR 5805-EPOC CNRS, Université Bordeaux 1, Arcachon, France; Paleontological Institute of Russian Academy of Science, United States of America

## Abstract

**Background:**

We examine the physiological and lifestyle adaptations which facilitated the emergence of ostracods as the numerically dominant Phanerozoic bivalve arthropod micro-benthos.

**Methodology/Principal Findings:**

The PO_2_ of modern normoxic seawater is 21 kPa (air-equilibrated water), a level that would cause cellular damage if found in the tissues of ostracods and much other marine fauna. The PO_2_ of most aquatic breathers at the cellular level is much lower, between 1 and 3 kPa. Ostracods avoid oxygen toxicity by migrating to waters which are hypoxic, or by developing metabolisms which generate high consumption of O_2_. Interrogation of the Cambrian record of bivalve arthropod micro-benthos suggests a strong control on ecosystem evolution exerted by changing seawater O_2_ levels. The PO_2_ of air-equilibrated Cambrian-seawater is predicted to have varied between 10 and 30 kPa. Three groups of marine shelf-dwelling bivalve arthropods adopted different responses to Cambrian seawater O_2_. Bradoriida evolved cardiovascular systems that favoured colonization of oxygenated marine waters. Their biodiversity declined during intervals associated with black shale deposition and marine shelf anoxia and their diversity may also have been curtailed by elevated late Cambrian (Furongian) oxygen-levels that increased the PO_2_ gradient between seawater and bradoriid tissues. Phosphatocopida responded to Cambrian anoxia differently, reaching their peak during widespread seabed dysoxia of the SPICE event. They lacked a cardiovascular system and appear to have been adapted to seawater hypoxia. As latest Cambrian marine shelf waters became well oxygenated, phosphatocopids went extinct. Changing seawater oxygen-levels and the demise of much of the seabed bradoriid micro-benthos favoured a third group of arthropod micro-benthos, the ostracods. These animals adopted lifestyles that made them tolerant of changes in seawater O_2_. Ostracods became the numerically dominant arthropod micro-benthos of the Phanerozoic.

**Conclusions/Significance:**

Our work has implications from an evolutionary context for understanding how oxygen-level in marine ecosystems drives behaviour.

## Introduction

**Figure 1:**
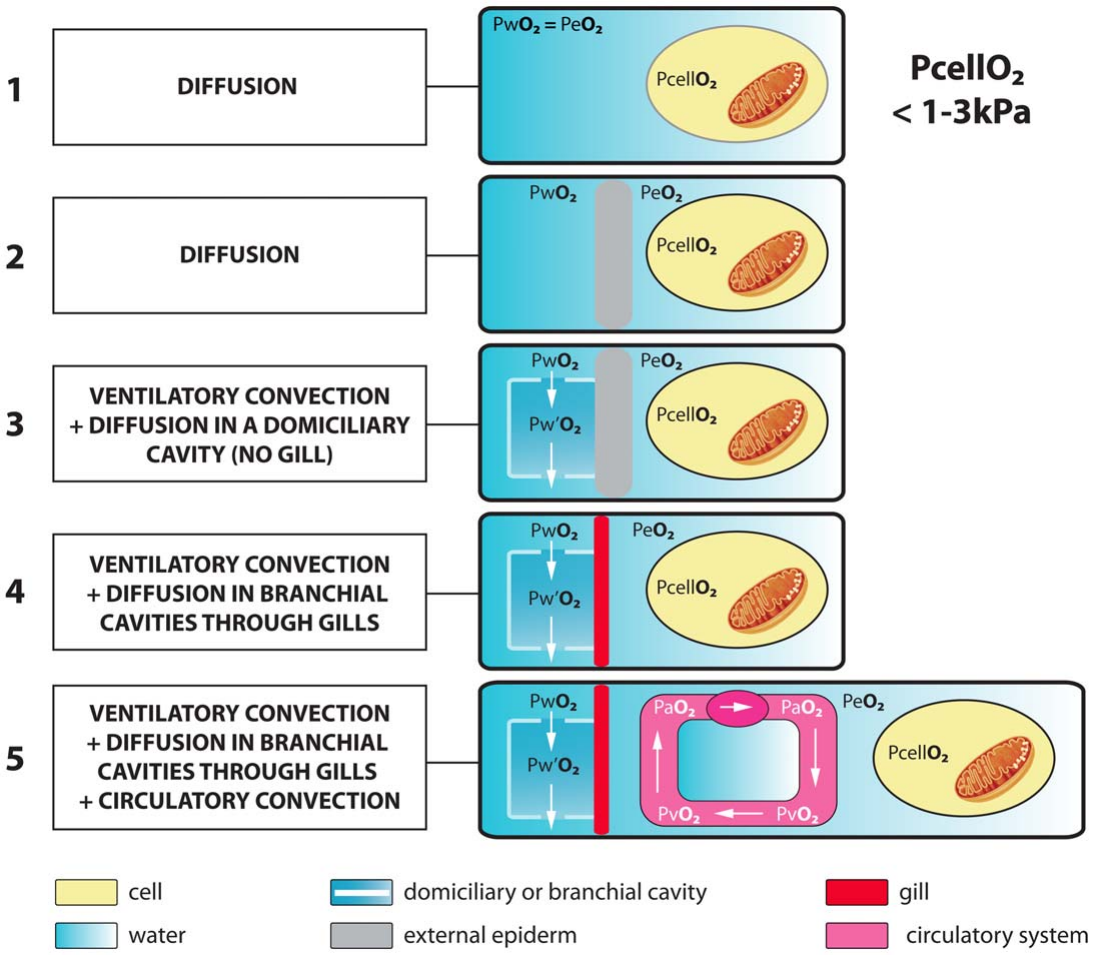
Respiration in water-breathers from simple gaseous diffusion (1) to ventilatory and circulatory convection (5). PwO_2_, PeO_2_, PcellO_2_, Pw′O_2_, PaO_2_, PvO_2_ correspond to the partial pressure of oxygen in water, in the extracellular medium, at cell level, within external chamber (e.g. branchial chamber), in arterial hemolymph, and in venous hemolymph. Today, the PO_2_ in the blood and tissues of water-breathing animals (see [4] for a review), is remarkably low, ranging between 1 and 3 kPa and is largely independent of ambient PO_2_. PcellO_2_ results from the equilibrium between O_2_ supply and O_2_ consumption (90% by mitochondria). PwO_2_>Pw′O_2_>PaO_2_>PeO_2_>PcellO_2_.

Molecular, geochemical and fossil evidence suggests that the first animals (eumetazoans) appeared during the late Precambrian [1] when atmospheric PO_2_ was between 8 and 15 kPa [2] compared to the modern 21 kPa, and the oceans were therefore less oxygenated than present. Recent geochemical studies indicate that atmospheric oxygen-levels increased during the late Precambrian, to reach high levels by the late Cambrian, perhaps as high as 30 kPa for several million years during the Furongian [3]. Whereas the earliest animals must have developed physiologies that allowed them to respire in benthic or pelagic environments where oxygen was less abundant, those that developed in the later Cambrian had to cope with similar or higher than present oxygen levels. Today, the PO_2_ in the blood and tissues of water-breathing aquatic animals (e.g. crustaceans, fish and molluscs; see [4], [5]), is remarkably low, ranging between 1 and 3 kPa and is largely independent of ambient PO_2_ ([4]; Figure 1). In mammalian tissues, the most frequently measured PO_2_ is also between 1 and 3 kPa [6]. Massabuau [4], [7] proposed that this widespread cellular oxygenation status using low oxygen level was a ‘memory’ of conditions that prevailed in the early stages of animal evolution when ambient oxygen levels were lower. The postulate is that maintaining cellular oxygenation at a very low level independent of the changes in ambient oxygenation that occurred throughout the Phanerozoic seems to have been a vital constraint to most aquatic animals, and is important in all animals for avoiding oxygen toxicity that would damage cell membranes and cellular machinery. Gaseous diffusion through the integument was probably sufficient for the smallest organisms relying on O_2_-metabolism to transport oxygen to their tissues, providing there was a sufficient gradient between the external medium and the tissues (PO_2_ in sea water >PO_2_ in tissues). This simple respiratory mode that requires no specialized organs may also have been used by larger sheet-like Ediacaran organisms [8] that possessed potentially large oxygen exchange surfaces (e.g. rangeomorphs). But this respiratory mode has strong limitations by imposing a relatively small distance between sea-water and tissues and a low or sluggish metabolism.

**Figure 2:**
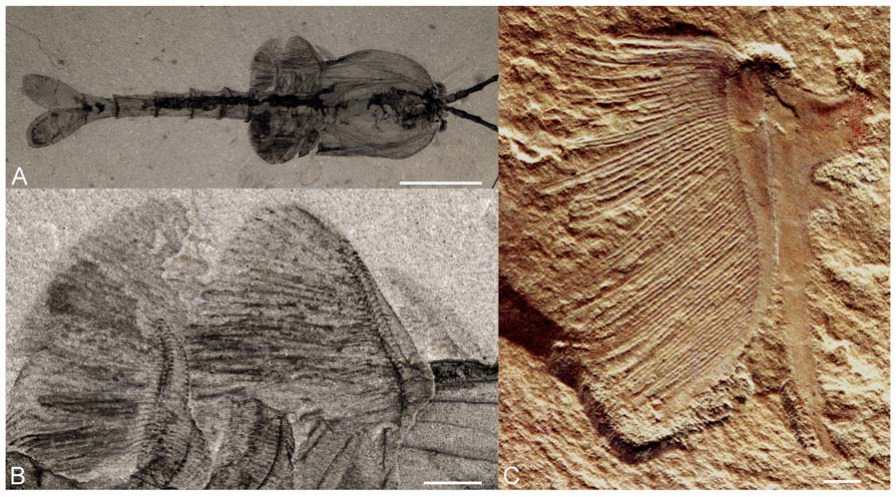
External respiratory features of Cambrian arthropods. A, B, *Waptia fieldensis* Walcott from the middle Cambrian Burgess Shale, British Columbia, Canada, USNM 138231 with trunk appendages bearing gill-like feature, dorsal view and close-up. C, *Naraoia longicaudata* Zhang and Hou from the early Cambrian Maotianshan Shale, Yunnan Province, China, NIGPAS 115315, biramous appendage with numerous gill-like filaments attached to exopodial branch. Scale bars: 1 cm in A and 1 mm in B and C.

The transportation of oxygen to the tissues via a circulating fluid (hemolymph) and a cardiovascular system (heart and vessels; Figure 1) and the increase of exchange surfaces via gills are two key-innovations that opened enormous opportunities to animal life: that of combining anatomical complexity, larger size and higher metabolic rates. The development of a sophisticated respiratory and circulatory system is particularly well exemplified by arthropods. This major phylum in present-day ecosystems was already well represented in the early Cambrian marine fauna, both in biodiversity and numerical abundance [9], and provides one of the best examples to study how respiratory strategies evolved in the Early Palaeozoic and how marine animals responded to the variations of oxygen level. Indeed, exceptionally preserved biota show that early arthropods already possessed sophisticated respiratory and circulatory systems as exemplified by the development of gills in many groups (see *Waptia fieldensis*, Figure 2) and also integument hemolymph networks.

Here we focus on three groups of Early Palaeozoic small bivalved arthropods with a well-documented fossil record: the phosphatocopids, the bradoriids and the ostracods and examine how they may have responded to changes in oxygen level at the beginning of the Palaeozoic Era. The three groups belong to the evolutionary history of crustaceans *sensu lato* (see [10]–[14]. More precisely in this paper we investigate environmental feedbacks caused by the spread of anoxia onto the marine shelf, and the rise of oxygen levels in seawater coupled to changes in seabed substrate that may have selected against bradoriid and phosphatocopid arthropods, but ultimately favoured the development of an ostracod micro-benthos.

## Results

**Figure 3:**
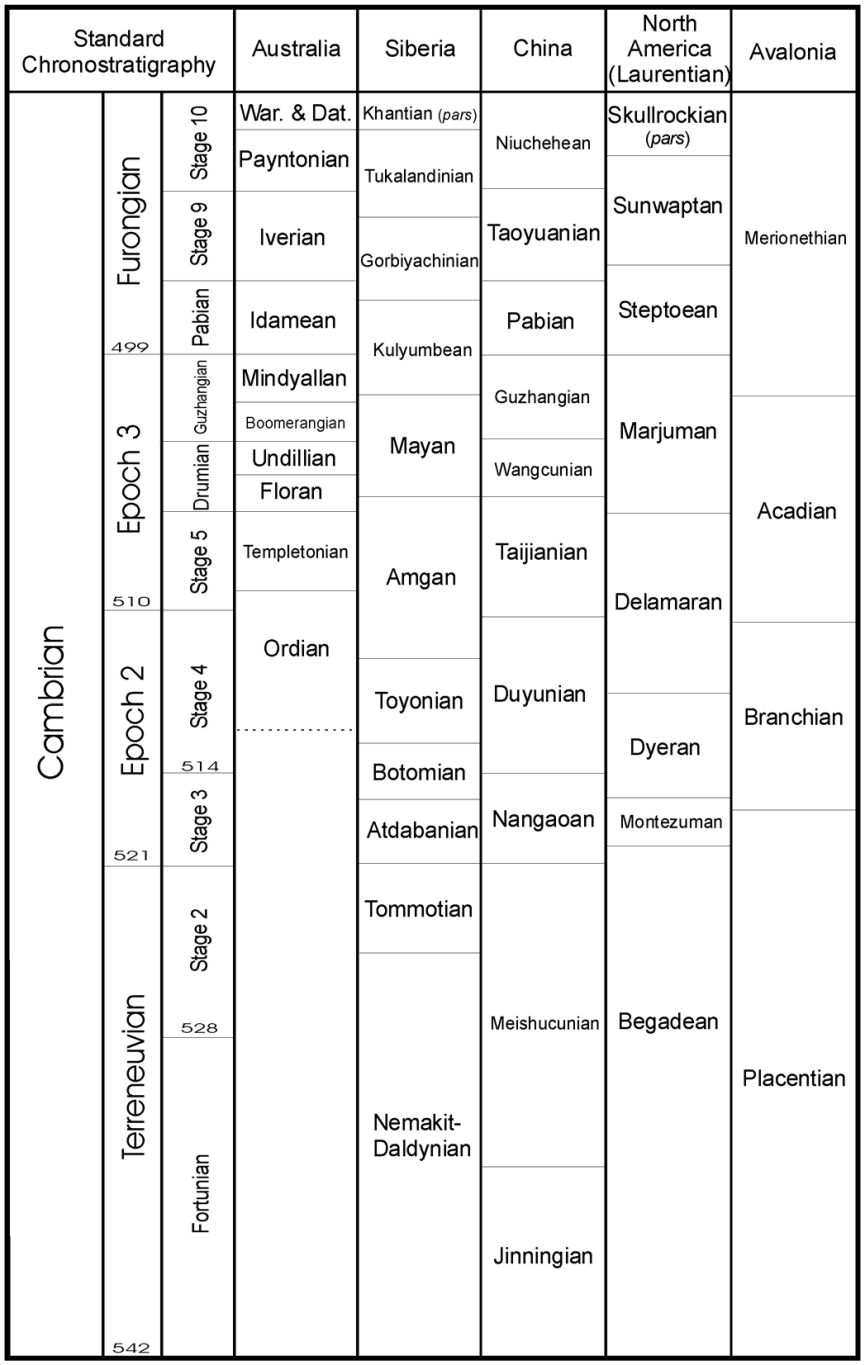
Chronostratigraphy for the Cambrian. Correlation of regional stratigraphies (modified from [58]) provides the key to understanding the bradoriid and phosphatocopid ranges reconstructed for Figure 4.

**Figure 4:**
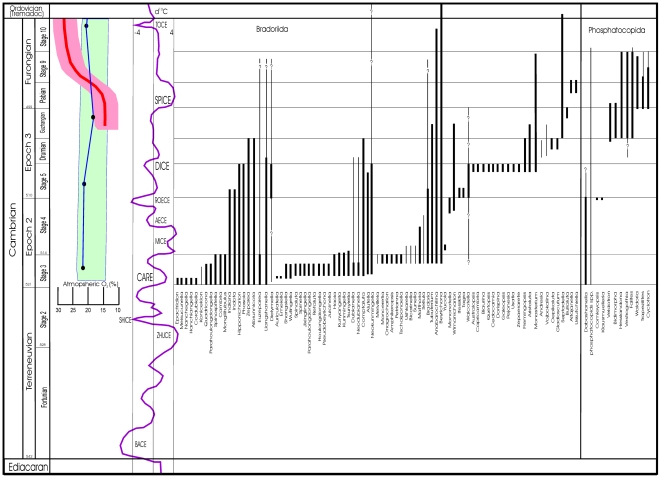
Temporal distribution of Cambrian arthropods conventionally assigned to the Bradoriida and Phosphatocopida. Bradoriid ranges are compiled from [57], [71], [72], [78] and references therein, representing a global dataset. Thick lines indicate definite ranges, whilst thin lines represent a questionable or imprecisely defined range. Only data from [46] and [71] are used to reconstruct the ranges of Bradoriida from China. The phosphatocopid data are from China [46], [77], Britain [43], Scandinavia and north Germany [13], [56], [63], [73], [79], [80], [81], [82], [83]: phosphatocopids are also known from the Antarctic [74], North America [66], Australia [84] and Kazakhstan [75]. The Borregård Member of Bornholm (equivalent to the Exsulans Limestone) was originally identified as the oldest horizon in Scandinavia with the phosphatocopids *Hesslandona*, *Vestrogothia*, *Bidimorpha* and *Falites* [79]. The Borregård Member is equivalent to the *P. gibbus* Biozone of uppermost Stage 5 [47], [83]. However, later the same author [82, p. 887] referred the material of [79] to the ‘Andrarum Limestone Breccia’, a horizon equivalent to the *P. forchhammeri* Biozone. Accordingly, we take the lower ranges of these phosphatocopids as Guzhangian. Our range for *Waldoria* includes material referred to *Falidoria* in [82]. We plot genera as a proxy for species diversity. This is reasonable given that most bradoriid genera contain only between 1 and 3 species. Exceptions to this include *Cambria*, *Liangshanella*, *Anabarochilina*, *Indiana* and *Hipponicharion*. For phosphatocopids, a multitude of species are referred to *Dabashanella*, but most of these are synonyms of *D. hemicyclica* [46]. Later Cambrian phosphatocopids including *Falites*, *Cyclotron*, *Hesslandona*, *Vestrogothia* and *Bidimorpha* all contain more than 3 species [78], emphasising the diversity of Guzhangian and Pabian phosphatocopid assemblages. Also shown are the major Carbon Isotope Excursions (CIEs) after [58], and oxygen levels reconstructed from [3]. For the latter, the red line represents the oxygen reconstruction (with error shown in pink envelope) of [3] and the blue line [with error envelope] is the Berner reconstruction quoted therein. In the text and figures we use the terms ‘early’, ‘middle’ and ‘late’ Cambrian informally to denote Cambrian Series 1 and 2 combined, Series 3, and Series 4 respectively. Note that some authors [13] would tentatively include *Epactridion*, *Dielymella*, *Liangshanella*, *Flemingopsis*, *Alutella*, *Oepikaluta* and *Gladioscutum* in the Phosphatocopida. For morphological reasons outlined in [57] we include these taxa within the Bradoriida. Abbreviations for CIEs are: BAsal Cambrian Carbon isotope Excursion (BACE); ZHUjiaqing Carbon isotope Excursion (ZHUCE); SHIyantou Carbon isotope Excursion (SHICE); Cambrian Arthropod Radiation isotope Excursion (CARE); MIngxinsi Carbon Isotope Excursion (MICE); Archaeocyathid Extinction Carbon isotope Excursion (AECE); Redlichiid-Olenellid Extinction Carbon isotope Excursion (ROECE); Drumian Carbon isotope Excursion (DICE); StePtoean Carbon Isotope Excursion (SPICE); Top of Cambrian Excursion (TOCE).

The temporal distribution of the bivalve arthropod micro-benthos in the Cambrian is shown through Figures 3 and 4. Here we use the morphology of the three main groups coupled with lithofacies distribution to suggest the mode of life and respiratory function of the animals. Though their record from the Cambrian is contentious, the most detailed discussion concentrates on the ostracods, for which physiological evidence from modern fauna is available and also because this is the only group that survived 500 million years of marine evolution to become a major Phanerozoic component of the micro-benthos.

### Ostracods

These are small (millimetre-length) aquatic crustaceans with a dorsally articulated bivalve carapace. The most numerically abundant arthropods in the fossil record, through their half billion-year history they have adapted to environments ranging from the abyssal depths of the oceans, to freshwater lakes and damp leaf litter. Earlier studies of the fossil record have documented the radiation of ostracods from the benthos into the marine zooplankton [15], [16] and from marine to freshwater environments [17], [18]. Whilst these adaptive radiations involved fundamental changes in ostracod morphology (e.g. the development of swimming appendages), and metabolism, the primary origin of an ostracod micro-benthos is obscure [19], [20]. Ostracods fulfil an important role in aquatic food webs as basal species feeding on primary producers and detritus from plants and animals, and are themselves predated by larger invertebrates and vertebrates. Some myodocopid ostracods live as micro-predators of other invertebrates in the water column, as scavengers [21], and even live as parasites on the gills of sharks [22]. The basal position of ostracods in ancient food webs is suggested by their occurrence in coprolites [23].

Using uniformitarian principles applied to carapace morphology the oldest benthic ostracods have been identified from early Ordovician strata (ca 485 Ma; [19], [24]). These early ostracods formed low-diversity (1 to 2 species) associations, are small (about 1 mm length), possess a bivalved dorsally articulated carapace and have lobal structures that are homologous to stratigraphically later fossil ostracods [25]. They are found associated with a range of marine benthos, which in their earliest occurrences [19], [20] includes trilobites and brachiopods. An ostracod micro-benthos was therefore established as a component of the earliest ‘Paleozoic fauna’ (*sensu* [26]) and these Tremadocian ostracods form the root of the major ostracod biodiversification event of the Middle Ordovician [25], [27]. Still earlier, putative ostracods are found in the Cambrian (see below).

**Figure 5:**
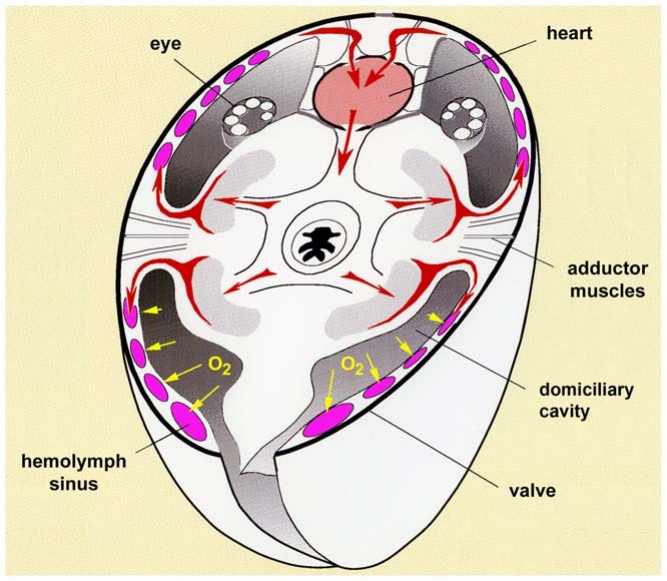
Respiration and circulation in Recent myodocopid ostracods. Simplified transverse section through anterior body and carapace: hemolymph route indicated by the red arrows. Hemolymph sinuses in pink (see Figure 6). Gaseous diffusion through the inner lamella of the carapace (see [51]) indicated by yellow arrows. The soft body, with or without gills or gas-exchange area, is bathing in the domiciliary cavity ventilated by the beating activity of two ventilatory plates.

**Figure 6:**
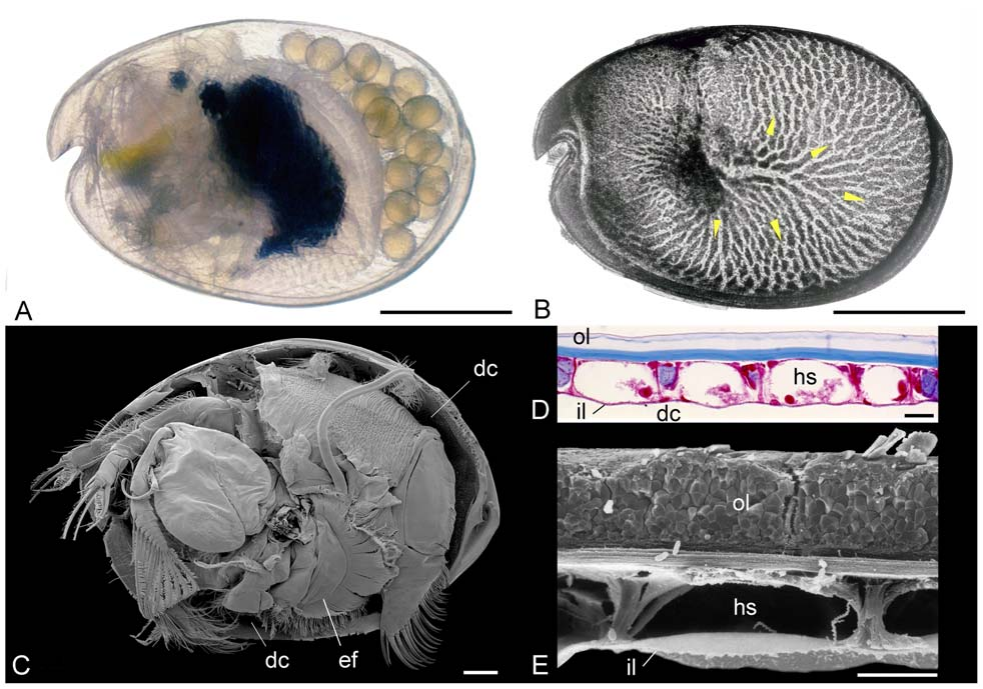
General morphology of Recent ostracods exemplified by myodocopids. A, B, *Vargula hilgendorfii* (Müller), left lateral view of live female carrying embryos in her domiciliar cavity (carapace translucent) and lateral view of left valve in transmitted light showing the gas-exchange area, an integumental hemolymph network (yellow arrows indicate hemolymph circulation). C, *Azygocypridina* sp. from New Caledonia, France. Scanning electron micrograph showing appendages, left valve removed, including the ventilatory plates (courtesy of Vincent Perrier). D, E, transverse section through the carapace of *Vargula hilgendorfii* showing hemolymph sinuses, stained microtome serial section and scanning electron micrograph, respectively (see [51], [76]). Scale bars: 1 mm in A–C, and 20 µm in D and E. ef, epipodial fan for ventilation; hs, hemolymph sinus; il, inner lamella; ol, outer lamella.

**Figure 7:**
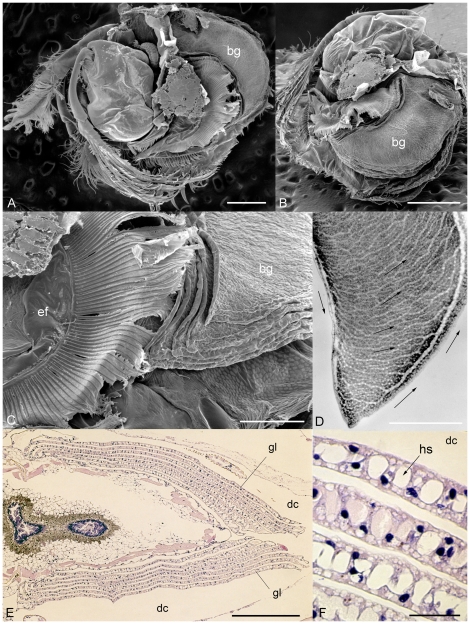
Recent ostracods with gills. *Leuroleberis surugaensis* Hiruta from Japan. A–C, scanning electron micrographs showing book gills in the branchial cavities, left lateral and posterior views. D, gill in transmitted light showing internal hemolymph sinuses. E, F, stained microtome serial sections, showing 8 pairs of gills on both sides of the soft body and detailed features through individual gills (e.g. hemolymph sinuses). Scale bars: 1 mm in A and B, 500 µm in D and E, 200 µm in C, and 50 µm in F. bg, book gill (integumental lamina attached to thoracic wall); ef, epipodial fan for ventilation; hs, hemolymph sinus in book gills.

Vannier and Abe [28] characterised ostracods as belonging to two morphological groups. ‘Body plan 1’ possesses bilateral symmetry, an ellipsoidal shape, an active mode of life (e.g. swimming) and a well-developed circulatory system and is typified by present and fossil myodocopid ostracods (Figures 5, 6). Some myodocopids have book gills as additional exchange surfaces attached in the latero-posterior part of their body (Figure 7). ‘Body plan 2’ corresponds to podocope ostracods *sensu lato* (podocopids and platycopids; see [29] for classification) with a ventral or lateral polarity ( = valve asymmetry), a heavily calcified shell, and is characterised by a crawling mode of life (mostly in the flocculent layer or sub-sediment surface) and no cardiovascular features (i.e. absence of a heart). Ostracods with ‘body plan 2’ often form a component of the meiofauna living interstitially between sediment grains in an environment which is both protected from predators (though some meiofaunal organisms feed on them), but rich in detrital, bacterial or algal food supply. These ostracods are small, typically in the range of 0.5 to 3 mm length [28]. Oxygen exchange occurs only via diffusion directly from the water through the integument of the carapace inner lamella and body. Although the water column where most Recent ostracods live is under or close to normoxic conditions (PO_2_ close to 21 kPa air-equilibrated water in open marine conditions), experimental studies [30] indicate that benthic ostracods migrate through the O_2_ gradient of the sediment in order to reach sediment layers where the PO_2_ of the water is much lower (ca 3–5 kPa). This low-oxygenation (hypoxic) level that occurs within a few millimetres below the water-sediment interface seems to be the preferential environment of numerous podocopid ostracods.

All ostracods possess a pair of ‘ventilatory appendages’ that beat rhythmically, creating currents between the inner side of the valves and the animal body over the main gas exchange tissue surfaces. Ventilation is produced by fan-like exopodial features with primary radiating bristles and closely packed secondary setae that offer resistance to water, typically on the 5^th^ limb of myodocopid ostracods. Importantly, the ventilatory movements are similar to what is observed in living green crabs *Carcinus maenas* or crayfish *Astacus leptodactylus* [30]–[32]. Laboratory experiments [30] performed with five podocopid genera (*Leptocythere*, *Cyprideis*, *Loxoconcha*, *Cytheropteron* and *Argilloecia*) indicate that podocopids lack regulatory mechanisms for adapting their ventilation to changes in water oxygenation in contrast to decapod crustaceans which can control their ventilation. Corbari et al. [30] propose that the migration of podocopid ostracods to poorly oxygenated (hypoxic) layers is a behavioural response to adjusting their tissue oxygenation to low-oxygen levels (of 1–3 kPa). Similar experiments were conducted with myodocopid ostracods [31]. Some myodocopids are pelagic (e.g. *Conchoecia*, [33]), others live at the water-sediment interface but show abilities to burrow into the sediment [34] and swim into the water column (e.g. nocturnal vertical migration of *Vargula*, [35]). Experiments with *Cylindroleberis mariae*, which possesses a system of gas transfer (ventilatory system and a cardiovascular system - dorsal heart and hemolymph network within the valves and well-developed book-gills), also demonstrated the inability of myodocopid ostracods to regulate their ventilatory and circulatory activities [31]. However, their behavioural response is more complex than for podocopids. During daytime they shelter within self-made nests (aggregates of sediment and mucous-substances) that create a hypoxic environment. Even more interestingly, this is the result of a behavioural and social strategy as groups of *Cylindroberis mariae* build the nests in which they are buried during daytime, re-breathing together in a confined environment. They become active during the night and swim into the water-column in normoxic water.

Although podocopids and myodocopids show major anatomical, physiological and behavioural differences, they both seem to respond in a similar way: both groups will avoid long-term exposure to normoxic conditions and migrate to low-oxygen conditions. Podocopids, which are sluggish and move slowly, do this continuously through the day. Myodocopids which are only active at night avoid normoxic water during daytime when they are at rest, buried in the top millimetres of sediment where oxygen is less abundant. If PO_2_ was low in the water column at the beginning of the Palaeozoic Era (PO_2_ at between 8 and 15 kPa, [2]), tiny early crustaceans similar to ostracods in terms of anatomy and physiology may have been able to live at the water-sediment interface or even in the water column without using migration through the sediment to reach low oxygen levels.

The oxygenation status at tissue and/or cellular level is not only dependant on the ambient PO_2_. It is also dependant on the cellular metabolism and the arterial vascularization and micro-vascularization. By definition, hypoxia occurs at the cellular level when oxygen input is not sufficient to fuel O_2_-consumption or when O_2_-consumption overreaches the oxygen supply. Thus, small planktonic ostracods (e.g. halocypridids) that live permanently within the water column, being active swimmers, possibly avoid high concentrations of O_2_ in their tissues not by hiding in low-oxygen niches but by “burning” O_2_ via a relatively high metabolism.

In marine and non-marine benthic aquatic environments most podocopid ostracods are found at and above the sediment-water interface and living interstitially within the sediment as meiofauna (e.g. [36], [37]). One specialised group of benthic podocope ostracods, the Platycopida, has developed a filter-feeding strategy (see [38] for a review). Corbari et al. [32] reassessed previous models of the oceanographic response of platycopids to widespread anoxia [38] in the light of experiments with *Cytherella*, a platycopid that seems to have developed various adaptations to resist hypoxia. *Cytherella* is quite different from other ostracods in that it has very thick valves and the ability to close them hermetically (using powerful muscles). Moreover, its activity level and ventilatory frequency is only half that of ostracods studied previously. When subjected to a decrease in oxygenation, it demonstrates the beginnings of ventilatory adaptation which is unknown in the other ostracod groups (podocopes, myodocopes), a strategy that may have enabled it to withstand intervals of seabed anoxia.

Preserved respiratory organs in fossil ostracods are rare, but examples from fossil Lagerstätten in the Silurian [39]–[41] and the Triassic [42] indicate that ancient myodocopids had book gills almost identical to those of their modern counterparts and fan-like exopodial features to ventilate their domiciliar cavity. The earliest ostracods of the Ordovician were small and it is highly probable that they lacked a specialized gas-exchange area (gills) and respired mainly by gaseous diffusion, as do most recent ostracods of similar body size. Indeed, the earliest ostracods possess no exoskeletal features that would indicate the presence of a hemolymph network.

### Phosphatocopids

**Figure 8:**
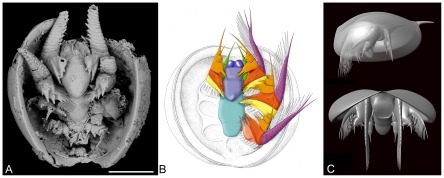
General morphology of phosphatocopid arthropods. A, *Hesslandona angustata* from the late Cambrian of western Hunan, China, ventral view of complete specimen with phosphatised appendages (see [77]). B, *Klausmuelleria salopiensis* Siveter, Waloszek & Williams from the Protolenus-Strenuella Limestone, early Cambrian (Series 2), Shropshire England, reconstruction of appendages in ventral view (courtesy of Dieter Waloszek, Ulm). Reconstruction is based on a specimen 0.34 mm long [11]. C, *Vestrogothia spinata* Müller, from the late Cambrian “Orsten” of Sweden, three-dimensional model, lateral and frontal views (courtesy of Joachim Haug). Scale bar: 100 µm.

These animals possessed a dorsally articulated bivalve shield which covered the entire body, the largest of which (in *Cyclotron*) approaches 6 mm in length [43], but more typically is 1 to 3 mm in length. Unlike ostracods, phosphatocopids lacked adductor muscles and may have been unable to control valve opening and closing, suggested by the many specimens preserved with ‘butterfly’ orientation (e.g. [12], [13], [43]; Figure 8). The lobate valves of some phosphatocopids (e.g. *Cyclotron*, [44]) are reminiscent of ostracods – the group to which they were first assigned [45], but their detailed soft anatomy, revealed for several different groups [13], indicates that phosphatocopids lacked modification of the first post-mandibular limb to form a maxillula and they are therefore not ostracods [12]. Most phosphatocopid crustaceans were small enough to enable oxygen diffusion through their cuticle (body and inner wall of carapace) and indeed none of the phosphatocopids have appendages bearing gills or integumental (circulatory system) networks.

Although phosphatocopids appear in Series 2 of the Cambrian (e.g. *Dabashanella*, [46]), their lithofacies distribution suggests they favoured areas of the seabed characterised by low-oxygen level. Phosphatocopids such as *Cyclotron*, *Vestrogothia*, *Trapezilites* and *Falites* dominated late Guzhangian and Furongian dysoxic benthic lithofacies, especially the olenid trilobite-bearing black shales of the English Midlands [43], [44], [47] and the organic-rich mudstones and limestone environments of the Alum Shales in Sweden [13], [48]. The Alum Shales are pyrite-rich, thinly laminated and show little evidence of bioturbation. They probably formed at a dysoxic or anoxic seabed ([48] and references therein) during an interval characterised by shoaling of anoxic waters onto the marine-shelf [49].

### Bradoriids

**Figure 9:**
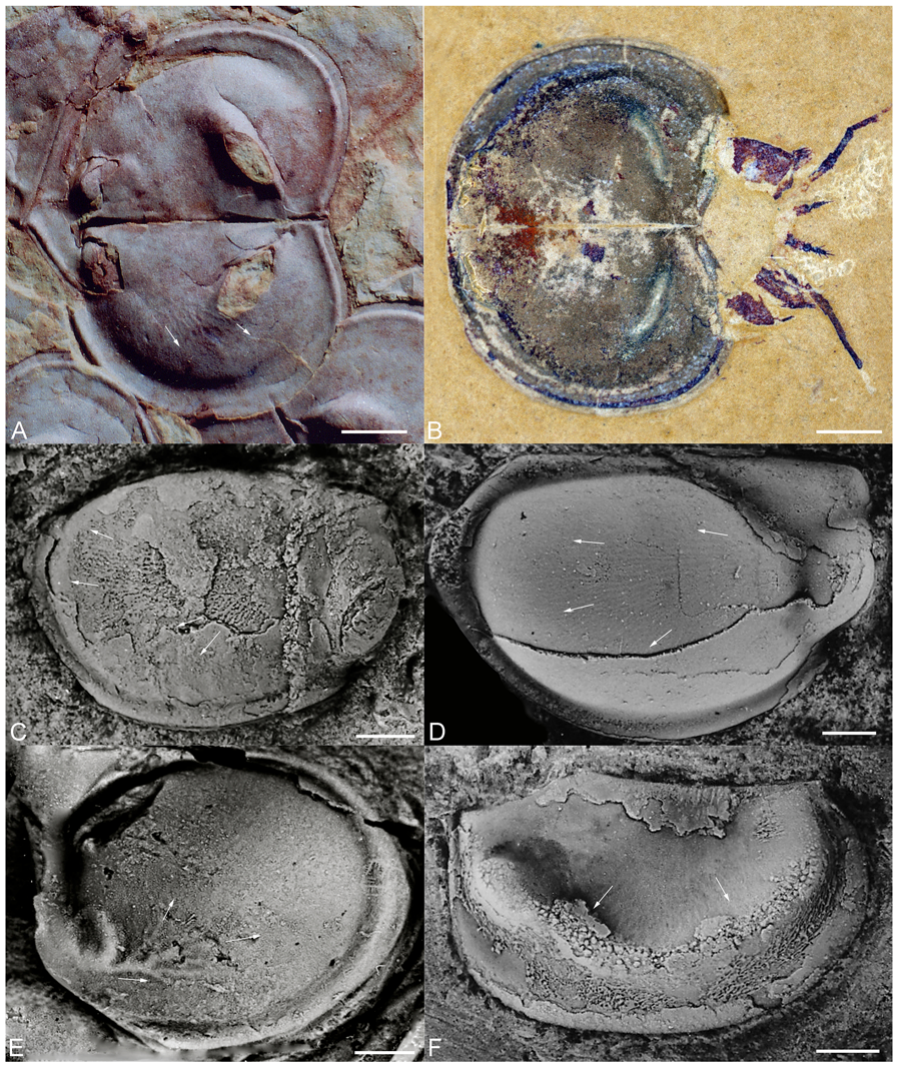
General morphology of bradoriid arthropods. A, B, *Kunmingella douvillei* (Mansuy) from the early Cambrian Maotianshan Shale, Yunnan Province, China, dorsal views of two specimens showing bivalved open carapace in “butterfly” position and posterior appendages, Chen Jun-Yuan's personal collections, Chengjiang and RCCBYU 10258, respectively (B, courtesy of Derek Siveter). C, *Anabarochilina* sp., middle Cambrian from Kazakhstan, PIN N4343/55, partly exfoliated right valve. D, *Anabarochilina primordialis* (Linnarsson), middle Cambrian from Västergötland, Sweden, SGU 8662, partly exfoliated right valve. E, *Tsunyiella gridinae* Melnikova from the early Cambrian (Atdabanian) of North Central Kazakhstan, PIN N4343/12, left valve, internal mould. F, *Cambria melnikovi* Ivanova from the early Cambrian (Atdabanian) of the eastern part of Siberia, Russia, PIN N2175/1, largely exfoliated left valve. Small white arrows indicate supposed integumental hemolymph networks (see [51]). All scale bars: 1 mm. For repositories of figured specimens see [14], [75].

These small arthropods possessed a dorsally folded bivalve shield of between 2 mm (e.g. in *Neokunmingella*) to 17 mm length (e.g. in *Petrianna*) for adults and this probably covered much of the body (Fig. 9). Long equated with the Cambrian record of ostracods [50], rare evidence from soft-anatomy shows that bradoriids lacked specialised post-antennal head appendages and are therefore not Eucrustacea [10], [14].

Like phosphatocopids, bradoriids lacked adductor muscles and many shields are preserved in butterfly' orientation with the valves gaping open. Bradoriid shields present striking evidence for the development of a complex cardiovascular system in this group [51], while the leaf-like exopodial branches attached to the appendages of some species such as *Kunmingella* suggest that bradoriids had additional gill-like features. Bradoriids were probably deposit feeders or scavengers of the numerous carcasses that were rotting on the seabed in epibenthic Cambrian ecosystems. Their basal position in Cambrian ecosystems is suggested by their occurrence in coprolite-like aggregates (e.g. Chengjiang, [52]) which suggests that they were predated by larger invertebrates. The facies distribution and shield morphology of bradoriids suggests that most of these animals adopted an active epibenthic lifestyle in well-oxygenated marine settings [10], [53]. Indeed, bradoriids are the most abundant animals in the epibenthic communities of the Burgess Shale [54], [55]. According to the initial hypothesis set out in the Introduction one must consequently suggest that they were active animals ‘burning’ O_2_ as fast as it diffused along the gradient of O_2_ partial pressure from ambient to cellular compartment. As a result of their relative environmental ranges, bradoriids and phosphatocopids tend to be mutually exclusive in the Cambrian rock-record, except where a few pelagic bradoriids [56], [57] are preserved in black mudstones at the seabed after death.

### Temporal patterns of micro-benthos diversity

The temporal distribution of the Cambrian bivalve arthropod micro-benthos is summarised through Figures 3 and 4 and is based on the assessment of faunas from all continents including Antarctica [57]. The dataset represents fossil materials collected over 140 years from hundreds of localities. We plot genera as a proxy for species diversity: this is a viable way to represent bradoriid diversity as genera typically contain only between 1 and 3 species [57]: exceptions to this are documented in the explanation to Figure 4. Early Cambrian phosphatocopid genera (e.g. *Comleyopsis*, *Klausmuelleria* and *Dabashanella* [12], [43], [46]) also contain only few species. Later Cambrian phosphatocopid genera are more species-rich, and *Bidimorpha*, *Cyclotron*, *Falites*, *Vestrogothia* and *Hesslandona* all contain greater than 3 species. Also shown on Figure 4 are the major Carbon Isotope Excursions (CIEs) of the Cambrian [58]. Some of these were associated with intervals of increased marine shelf anoxia and black shale deposition [49], [59]. The peak diversification of Bradoriida occurred during the Cambrian Arthropod Radiation Event (‘CARE’ *sensu* [58]). During this interval species radiated into a wide range of high and low latitude shelf sea environments [57] including deep shelf [60] and marginal marine settings [53], [61]. Following the ‘CARE’ event bradoriids underwent major extinctions, during late Stage 3 (Atdabanian-Botomian boundary, and early to mid Botomian) with the loss of at least 25 genera and the demise of the entire Kunmingellidae and most of the Cambriidae Family (Figure 4). Taxa that survived these extinctions include several long-ranging genera such as *Liangshanella*, *Beyrichona* and *Anabarochilina*. Further originations of essentially short-ranging bradoriid taxa occurred during late Stage 4, Stage 5 and the Drumian (Figure 4). Bradoriid diversity declined significantly from late Stage 5 to the Drumian boundary (e.g. the loss of long-ranging genera such as *Hipponicharion*), and at the Drumian-Guzhangian boundary (Figure 4). As a result, bradoriids become extremely rare in late Cambrian strata worldwide, with the exception of one group, the svealutids [57] (Figure 9). The longevity of svealutids such as *Anabarochilina* (Figure 4) was perhaps facilitated by their ability to disperse widely [57]. One bradoriid taxon, *Beyrichona* (represented by the very long-ranging species *B. triceps* [43]), ranged into the earliest Ordovician but disappeared from the rock record in the *Angelina sedgwickii* Biozone at the end of the Tremadocian [43].

**Figure 10:**
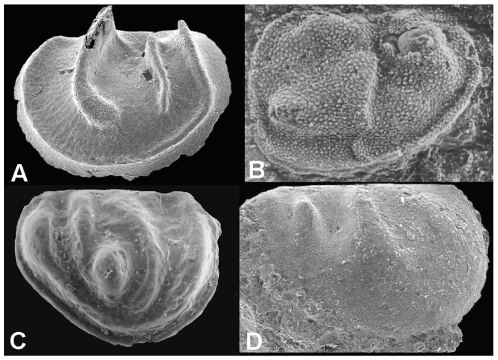
Early ostracods from the Cambrian and Ordovician. A, *Kimsella luciae* Salas, Vannier & Williams, lateral view of right valve. Tremadocian, lower part of the Parcha Formation, Abra de Sococha Section, Province of Salta, Argentina, CORD-MP 11186. B, *Altajanella costulata* Melnikova, lateral view of right valve. Late Cambrian, Tandoshka Formation, Gorny Altay, PIN N4346/1. C, *Vojbokalina magnifica* Melnikova, left lateral view of carapace. Middle Cambrian, Leningrad Region, Russia, PIN N4341/6. D, *Nanopsis coquina* Salas, Vannier & Williams, lateral view of left valve. Tremadocian, Upper Member of the Coquena Formation, Quebrada Chalala Section, Cordillera Oriental, Argentina, CORD-MP 11179. Scale bars are: 100 µm. For repositories of figured specimens see [20].

The latest originations of bivalved arthropods traditionally assigned to the Bradoriida occurred during the Guzhangian and Pabian, and include *Septadella*, *Altajanella*, *Bullaluta* and *Uskutchiella* (Figure 4). Of these, *Bullaluta* appears assignable to the Svealutidae and *Uskutchiella* to the Hipponicharionidae [57]. The two other taxa are characterised by small size (1–2 mm) and lack evidence from their shield morphology for the development of a cardiovascular system. The lobation of their shields is indistinguishable from those of early Ordovician ostracods (Figure 10) and they do not occur in dysoxic black shale lithofacies with phosphatocopids. They have been mooted as the earliest ostracods [20], [57].

Phosphatocopids also appear during the ‘CARE’ (Figure 4), but have a sporadic distribution in the rock record. In southern Britain, for example, where the Cambrian record has been intensively studied for 150 years they are known only from the Comley Limestones of Shropshire [62] and the black shales of South Wales and the English Midlands [43], [44]. Phosphatocopids are most common at horizons associated with dysoxic environments. Peak abundances and diversity of Phosphatocopida are associated with sedimentary deposits of the Guzhangian and Pabian (Figure 4), the latter originations (e.g. *Trapezilites*, *Cyclotron*) being stratigraphically coincident with the beginning and peak of the SPICE event [63], [64]. In England, Wales and Newfoundland, this interval is associated with black shale deposition that yields species of *Cyclotron*, *Vestrogothia*, *Falites*, *Veldotron*, *Trapezilites* and *Waldoria* [43], [44] in lithofacies which are devoid of benthic bradoriids. Similarly diverse phosphatocopid assemblages are known from the Alum Shales of Scandinavia, in the same stratigraphical interval (‘phophatocopine lithofacies’, see [63]), from a succession that also includes the earliest chemotrophic (olenid) trilobites that signal very low oxygen seabed conditions [65]. The Alum Shales environment appears to have varied between seabed anoxia and slightly more oxygenated conditions (dysoxic or hypoxic), with phosphatocopids occupying the latter environments. Phosphatocopid diversity declined during Stage 9 and they disappeared from the rock record a few million years after their peak diversity.

## Discussion

In this section we examine the feedback mechanisms that may have driven changes in bradoriid and phosphatocopid diversity. We begin by assessing the level of Cambrian seawater oxygen. We then examine how changes in marine oxygen may have led to the demise of bradoriids and phosphatocopids, but favoured a third group of arthropod micro-benthos, the Ostracoda.

### Cambrian seawater oxygenation

There is a consensus that oxygen levels were rising from the late Precambrian through into the Cambrian [2], from perhaps an initial value of between 10 and 15 kPa to values perhaps as high as 30 kPa by the late Cambrian [3]. Although surface ocean waters may have been more oxygenated, the deeper waters of Cambrian oceans probably remained anoxic [49]. Thus, overall increasing oxygen-levels in the oceans were punctuated by a series of ocean anoxic events, in part caused by intervals of marine transgression in which deep marine anoxic waters shoaled onto the shelf resulting in the accumulation of black shales [49], [59]. Many of these events are equated with marked carbon isotope excursions that are associated with marine extinction events [58]. Salzmann et al. [3] used a carbon and sulphur isotope mass balance model to show a dramatic increase in oxygen-levels through the interval from 502 to 496 Ma (through the Guzhangian – Pabian Stage boundary) that encapsulates the interval of the SPICE positive CIE (Figure 4). The SPICE event at the base of the Furongian Series likely represents an interval of increased organic carbon burial beneath euxinic ocean waters that extended onto the shelf. The positive carbon (δ^13^C_carb_) and sulphur (δ^34^S_CAS_ – carbonate associated sulphur) isotope excursions associated with this event would have been associated with net O_2_ production [3]. Beginning with initial values of PO_2_ (taken from the Berner model), Salzmann et al. [3] calculated peak PO_2_ at about 30 kPa (Figure 4). Oxygen-levels may have remained high for several million years [3] and this build-up of oxygen would eventually have resulted in more oxygenated marine shelf waters. The build-up of atmospheric oxygen during the peak of the SPICE event would have produced a negative feedback mechanism on the spread of anoxic waters that had initiated the global mass extinction at the beginning of SPICE [3].

### The Early Cambrian dominance of bradoriids

Bradoriids originating during the ‘CARE’ mostly possessed large shields that exhibit clear evidence of hemolymph networks and a cardiovascular system [51] (Figure 9). These early Cambrian bradoriids appear to have adopted an active mode of life within (normoxic Cambrian-seawater) oxygenated marine shelf waters. The Chengjiang bradoriid *Kunmingella douvillei* [10], [14] and the Burgess Shale *Liangshanella* (JV, unpublished) both possess biramous appendages. The endopods of these limbs had an obvious crawling function suggesting that the animals lived in contact with the seabed. The exopods are leaf-like and although no microstructures are preserved (e.g. hemolymph sinuses) the exopods may have functioned both for respiration and swimming. Bradoriids are likely to have been highly mobile arthropods the contrary of slow moving animals, moving rapidly at the water sediment-interface or just above it.

There is abundant evidence of gills in early Cambrian arthropods (e.g. lamellae and filaments on every appendage; Figure 2) and of a network of branching sinuses adjacent to the inner side of the shield of many Cambrian arthropods (unpublished information of JV). Development of advanced cardiovascular systems may have been the only way for relatively large animals to develop highly mobile lifestyles that required sufficient oxygen, particularly if early Cambrian shelf seas had relatively reduced oxygen-levels (10 kPa air-equilibrated water). A hemolymph circulation powered by a heart seems to have enabled more active behaviour in the water column among a variety of arthropods including the bradoriids.

The ‘CARE’ radiation of bradoriids into a range of marine shelf environments during early Stage 3 (Atdabanian; Figures 3, 4) was curtailed during mid to late Stage 3 with the extinction of at least 25 genera (Figures 3, 4). These extinctions may correlate with widespread black shale deposition on several continents, commencing as early as the late Atdabanian in Australia and China (59), and associated with shelf anoxia and a positive carbon isotope excursion that has been christened the Sinsk event [59]. The early to mid Botomian interval (Figure 3) is characterised by a rapid decline in archaeocyathans, Tommotians and some trilobites. For organisms like bradoriids with an advanced cardiovascular system and an active mode of life the spread of deep-ocean anoxic waters onto the marine shelf would have been lethal. As the majority of earlier bradoriids are interpreted to have occupied epibenthic niches [66], and many such as *Kunmingella*, *Auriculatella* and *Shangsiella* possessed limited intercontinental dispersal capabilities – at generic level they are strongly provincial [57], they may have been unable to escape anoxic seabed waters. In such a scenario the low PO_2_ gradient between the external seawater medium and the bradoriid tissues would have rendered respiratory organs inefficient, especially also because they lack ventilatory plates to efficiently ventilate their gills. Nevertheless some bradoriid taxa that originated during Stage 3, such as *Beyrichona* and *Liangshanella* survived these extinctions (Figure 4) and became long-ranging taxa. In the case of the svealutid *Liangshanella* its survival may have been facilitated by species of this genus adopting an active swimming lifestyle that enabled them to escape anoxic dead zones. Species of the epibenthic *Beyrichona* were amongst the most widely dispersed of Cambrian bradoriids occurring in both the Redlichiid and Olenellid trilobite realms and from low to high latitudes [57], a range that would have facilitated survival (in oxygenated refugia) during periods of widespread ocean anoxia.

Extinctions and originations of bradoriids continued through Cambrian stages 4 and 5, but bradoriid diversity appears to have been significantly curtailed for a second time at the Stage 5-Drumian boundary (Figure 4), an interval associated with marine transgression [63] and black shale deposition [47], [63]. Thus, in the black shales of the Menevian Group of south Wales [47] benthic bradoriids are absent, excluded by dysoxic seabed conditions, though pelagic svealutids are present [43]. In the Scandinavian succession bradoriid-bearing strata referable to the *B. oelandicus* trilobite biozone of Stage 5 are succeeded by black shales of the *P.gibbus* biozone just below the top of Stage 5. Further bradoriid extinctions occurred at the Drumian-Guzhangian boundary, including the loss of several long-ranging taxa such as *Comptaluta* and *Phasoia* (Figure 4). Thus, by the onset of the Pabian Stage SPICE event, which was also associated with widespread shelf sea anoxia [3], [49], [64], virtually all of the large, earlier Cambrian bradoriids were extinct. The single group of large bradoriids which remain widespread in Pabian age rocks, the pelagic svealutids (Figure 9C–E), possessed well-developed cardio-vascular systems that supported an active free-swimming lifestyle and lived in well-oxygenated waters above areas affected by anoxia.

Benthic bradoriids remained rare in the late Cambrian, as did all small bivalved arthropod micro-benthos [57]. This may reflect: 1) the initial widespread seabed anoxia during the SPICE event [49]; 2) more oxygenated surface marine waters following this interval; and [3] the physiological inability of benthic bradoriids to avoid seawater O_2_ levels well above those in which the early Cambrian bradoriid fauna had initially evolved. Bradoriids were too large to occupy the interstices between sediment grains (as ostracods do to escape normoxic conditions) and they also lacked the ability to tightly close their shields, thus negating their ability to control oxygen levels in the waters immediately adjacent to their respiratory organs.

### Phosphatocopids and dysoxic seabeds

Even in those successions most intensively studied for bivalved arthropod micro-benthos, the occurrence of phosphatocopids is sporadic [43], [46], [66]. However at certain horizons, particularly in late Guzhangian and early Furongian black shales [44], and organic-rich limestones [48], [63] phosphatocopids became an abundant component of the benthos. These ‘phosphatocopine facies’ lie adjacent environments characterised by pyrite-rich shales, chemotrophic olenids or pelagic agnostids [65], in sedimentary deposits formed in low-oxygen or anoxic seabed conditions [48], [63], [65] that excluded benthic bradoriids with cardiovascular systems adapted for oxygenated waters (see above). As phosphatocopid arthropods lacked a cardiovascular system [13], they must have possessed a metabolism that favoured dysoxic benthic conditions and their diversity and abundance peaked during the widespread shelf anoxia of the SPICE event [49]. Their lower abundance in post *Olenus* Biozone strata [43], and disappearance from the fossil record in the late Cambrian (*peltura* Biozone, ca 490 Ma) may be linked to the elevated oxygen-levels of late Cambrian shelf seas that kept deep water anoxia at bay [3].

### The ostracod micro-benthos

Post-DICE event (Figure 4) shelf-marine benthic environments that were not oxygen-poor contained an impoverished small arthropod micro-benthos. This micro-benthos became even poorer during and after SPICE (Figure 4). In contrast, the peak diversity of phosphatocopids in benthic environments occurred during SPICE (Figure 4), with the spread of anoxic waters onto the shelf. In the interval following DICE, originations in the arthropod micro-benthos are characterised by tiny (millimetre-scale) sized animals (Figure 10), much smaller than the bradoriids that originated during ‘CARE’. These small animals include *Altajanella* and *Vojbokalina* that possess shields which appear to conform to ostracod ‘body plan 2’ of Vannier and Abe [28], with a small elliptical shape and apparently no sophisticated respiratory and circulatory systems: at least the latter are not reflected in the morphology of the shield (Figure 10). These small bivalved arthropods of the post-DICE Cambrian world were confronted with four major environmental changes: 1) the spread of marine anoxia onto the shelf during SPICE; 2) more oxygenated shallow marine waters post-SPICE [3]; 3) open ecospace to colonise (the absence of Bradoriida in epibenthic niches); and 4) substrate change as microbial mats retreated [67]. While phosphatocopid arthropods disappear from the Cambrian record after the *peltura* trilobite Biozone (Stage 10; see Figure 4) and may have been unable to adapt to rising seawater PO_2_, even by retreating into sediment, organisms with a range of more ‘oxygen-tolerant’ life strategies and anatomies (e.g. greater resistance to O_2_ diffusion; more active O_2_ consumption in tissues; retreat to zones of lower water oxygenation; ability to regulate the O_2_ content of waters adjacent respiratory tissues) may have been selectively favoured. Ostracods tolerate normoxic sea conditions and even during intervals of elevated oxygen can retreat to the interstices of sediment where hypoxic conditions prevail [30]. The earliest ostracods may have originated in very shallow marine waters that remained oxygenated even during the maximum spread of seabed anoxia at the beginning of SPICE. Certainly the earliest Tremadocian ostracods from rocks 15 million years later than SPICE are found dominantly in shallow shelf marine environments that appear to have been well-oxygenated [20], [24]. *Vojbokalina*, *Septadella* and *Altajanella* are therefore plausible early ostracod micro-benthos.

In tandem with changes in marine oxygenation, there were also significant changes in marine substrate evolution that may have benefited a burrowing (ostracod) micro-benthos. During the Cambrian, seabed sediments became increasingly bioturbated, for example by worms [68], which redistributed organic matter through the sediment and thus provided new food sources and new habitats (micro-environments). These conditions facilitated the colonization of sediment by a range of meiofauna [69], though these did not, initially, include ostracods. In tandem with the increasing availability of oxygen, and greater seabed bioturbation, the distribution of microbial mats was curtailed in the late Cambrian [67]. The “substrate revolution” [70] may have enabled more easy penetration of seabed environments by small organisms inhabiting the flocculent layer, and facilitated feedbacks that favoured the evolution of small size. Certainly ostracods with their small size and well developed musculature for closing the protective shield were amongst the first successful colonizers of these niches. In addition, if their physiology was similar to that of recent podocopid ostracods, a substrate with oxygen levels lower than those in the water column would have been the most suitable habitat for them to colonise. Bradoriids were excluded from such environments by their large size and life attitude with the two valves widely open - poorly consistent with digging habits, and by their absence of musculature to control shield closure. Phosphatocopids were excluded by elevated levels of oxygen that were hostile to a physiology adapted for very low-oxygen seabed conditions.

The arthropod micro-benthos remained impoverished throughout the post-SPICE interval (Figure 4) even when widespread marine shelf anoxia retreated. This poor diversity may have been compounded by the elevated PO_2_ levels of the late Cambrian that may have restricted the environmental distribution of ostracods, despite their ability to retreat from normoxic waters. For those organisms with more limited mechanisms to control the oxygen levels of their body tissues this may have been catastrophic.

### Conclusions

The fossil record of Cambrian bivalve arthropod micro-benthos suggests strong feedbacks between marine oxygen level and evolution. Benthic bradoriids evolved well-developed cardiovascular systems that favoured their colonization of oxygenated marine shelf waters but possibly in a fairly narrow range of O_2_-levels (between 10 and 15 kPa). Their biodiversity declined during bouts of Cambrian marine shelf anoxia and may also have been curtailed by elevated late Cambrian oxygen-levels that dramatically increased the PO_2_ gradient between seawater and bradoriid tissues. Phosphatocopids responded to Cambrian anoxia differently, reaching their peak in environments characterised by seabed dysoxia. As late Cambrian marine shelf waters became well oxygenated, phosphatocopids went extinct. Changing seawater oxygen-levels and the demise of much of the seabed bradoriid micro-benthos may have favoured a third group of Early Palaeozoic arthropod micro-benthos, the ostracods. These animals have lifestyles and strategies which make them tolerant of changes in seawater oxygen-level, able to close their valves tightly and able to burrow into sediment both to utilise food sources there, escape predation and also occupy zones of lower (hypoxic) oxygen conditions. It was the ostracods which went on to become the numerically dominant bivalve arthropod micro-benthos of the Phanerozoic, radiating into a vast range of aquatic environments from the Ordovician onwards.
